# *Nigella sativa* supplementation and non-alcoholic fatty liver disease: A systematic review of clinical trials

**DOI:** 10.22038/AJP.2022.20060

**Published:** 2023

**Authors:** Abbas Mohtashamian, Armin Ebrahimzadeh, Zahra Shamekhi, Nasrin Sharifi

**Affiliations:** 1 *Research Center for Biochemistry and Nutrition in Metabolic Diseases, Basic Science Research Institute, Kashan University of Medical Sciences, Kashan, Iran*; 2 *Sepidan Bagherololoom Higher Education College, Shiraz University of Medical Sciences, Shiraz, Iran*

**Keywords:** Nigella sativa, Non-alcoholic fatty liver disease, Clinical trials, Systematic review

## Abstract

**Objective:**

Based on the results of previous studies, the effects of *N. sativa* on some of the non-alcoholic fatty liver disease's (NAFLD) biomarkers were positive; however, there were conflicting results regarding other variables. Therefore, the present systematic review of clinical trials was designed to clarify whether *N. sativa* effectively prevents the progression of NAFLD.

**Materials and Methods:**

A search of four databases (Scopus, PubMed, Medline, and Google scholar) was conducted to identify the clinical trials that assessed the effects of *N. sativa* supplementation on NAFLD. The outcome variables of interest were biomarkers of hepatic steatosis, liver enzymes, insulin resistance, and inflammation.

**Results:**

Overall, four randomized clinical trials (RCTs) were included. In three studies, hepatic steatosis grade decreased significantly after *N. sativa* supplementation. Serum levels of liver enzymes reduced significantly in three of four included trials. In the only study that examined the effect of *N. sativa* on insulin resistance parameters, all variables related to this factor were significantly reduced. In two included studies that measured biomarkers of inflammation, the serum levels of tumor necrosis factor α (TNF-α), high-sensitive C-reactive protein (hs-CRP), and interleukin 6 (IL-6) decreased significantly after intaking *N. sativa* supplements.

**Conclusion:**

Although the efficacy of *N. sativa* on liver enzymes and the grade of hepatic steatosis was reported in some of the included studies, more well-designed clinical trials are needed to determine the definitive effects of *N. sativa* on NAFLD. The present study provides suggestions that help to design future studies in this field.

## Introduction

Non-alcoholic fatty liver disease (NAFLD) is excessive fat accumulation in the liver without alcohol use (Mokhtari et al., 2017 ▶). NAFLD prevalence in the Middle East and Africa is 31.8 and 13.5%, respectively (Younossi et al., 2019 ▶). NAFLD includes various stages such as liver steatosis, non-alcoholic steatohepatitis (NASH), liver fibrosis, and cirrhosis. The fundamental risk factors of heart disease, such as hyperlipidemia, obesity, and diabetes mellitus, are related to the pathogenesis of NAFLD (Cicero et al., 2018 ▶). Weight loss, adherence to a healthy diet, and increasing physical activity are the only established strategies to prevent and treat NAFLD (Argo et al., 2009 ▶; Keating et al., 2012 ▶; Lindenmeyer and McCullough, 2018 ▶; Mummadi et al., 2008 ▶; Promrat et al., 2010 ▶). However, research is ongoing to find drugs or supplements that may help in managing NAFLD (Cicero et al., 2012 ▶; Hashempur et al., 2015 ▶; Hosseini et al., 2016 ▶; Samani et al., 2016 ▶; Sharifi and Amani, 2019 ▶; Sharifi et al., 2014 ▶).

Recently, herbal medicines have gained attention as adjuvant therapies in various diseases related to metabolic syndrome, such as diabetes (Yeh et al., 2003 ▶), hypertension (Chen et al., 2015 ▶), obesity (Hasani-Ranjbar et al., 2009 ▶; Sui et al., 2012 ▶), and hyperlipidemia (Hasani-Ranjbar et al., 2010 ▶). This is due to the antioxidant and anti-inflammatory properties of herbal compounds that allow them to prevent and reduce the complications of chronic diseases (Ben El Mostafa and Abdellatif, 2020 ▶). One of the herbs that have a beneficial effect on metabolic syndrome components such as hypertension, hyperlipidemia, and insulin resistance is *Nigella sativa* (*N*. *sativa*)(Benhaddou-Andaloussi et al., 2011 ▶; Muneera et al., 2015 ▶; Sahebkar et al., 2016 ▶). *N. sativa* is an annual flowering plant in the family Ranunculaceae and is native to South and West Asia. *N. sativa* is also known by other names such as black cumin, fennel flower, black seeds, nutmeg flower, black caraway, and kalonji (Hussain et al., 2017 ▶). *N. sativa* seed oil and powder have been consumed for a long time for treating various diseases (Hussain et al., 2017 ▶). In addition, *N. sativa* has antioxidant, anti-inflammatory, anti-fibrotic, and anti-bacterial properties that have advocated researchers to examine its treatment effects on NAFLD (Amizadeh et al., 2020 ▶; Ben El Mostafa and Abdellatif, 2020 ▶; Hadi et al., 2016 ▶). Several randomized clinical trials (RCTs) have been conducted until now to evaluate the effects of *N. sativa* on fatty liver disease (Darand et al., 2019a ▶; Darand et al., 2019b ▶; Hosseini et al., 2018 ▶; Hussain et al., 2017 ▶; Khonche et al., 2019 ▶; Rashidmayvan et al., 2019 ▶).

In these studies (Darand et al., 2019b ▶; Hosseini et al., 2018 ▶; Hussain et al., 2017 ▶; Khonche et al., 2019 ▶; Rashidmayvan et al., 2019 ▶), the effects of *N. sativa* supplementation on various factors such as the grade of fatty liver and steatosis, liver enzymes, biomarkers of glucose metabolism, inflammatory factors, lipid profile, anthropometric indices, and blood pressure have been evaluated. However, there are conflicting results on the effects of *N. sativa* regarding some of the NAFLD biomarkers. Therefore, the present systematic review of clinical trials was designed with the following objectives: 1. To discuss whether *N. sativa* effectively prevents steatosis and fibrosis progression in NAFLD; 2. To compare the findings of the clinical trials in terms of the effectiveness of *N. sativa* on the secondary outcomes of NAFLD such as fasting blood glucose (FBG), serum insulin, homeostatic model assessment for insulin resistance (HOMA-IR), lipid profiles, blood pressure, inflammatory biomarkers and anthropometric indices; and 3. To provide suggestions for future well-designed studies in this field.

## Materials and Methods

**Search strategy**

A systematic review of the literature was conducted for uncontrolled clinical trials, randomized controlled trials (RCTs), and non-randomized interventions, which indicate the results of *N. sativa* supplementation on hepatic steatosis, liver enzymes, lipid profiles, and blood sugar or insulin sensitivity in patients with NAFLD. It was considered sufficient for studies to report the NAFLD diagnostic methods based on one or more of the following: (1) ultrasound; (2) blood concentrations of alanine aminotransferase (ALT) and aspartate aminotransferase (AST); (3) histological examination of biopsies; (4) computed tomography (CT); and (5) proton magnetic resonance spectroscopy (MRS). 

The primary outcomes of interest included changes in serum levels of liver enzymes and hepatic steatosis and fibrosis assessed by liver biopsy, CT, histological indicators of inflammation, ultrasound, and MRS. Fasting blood glucose (FBG), serum insulin, homeostatic model assessment for insulin resistance (HOMA‐IR), lipid profiles, blood pressure, inflammatory biomarkers, and anthropometric indices were considered the secondary outcomes. 

A systematic literature search was conducted separately by two researchers (AM & AE), from inception to January 21-2021, to assess and select studies of *N. sativa* supplementation on NAFLD.

Some databases, including Scopus, PubMed (Medline), and Google Scholar, were applied to perform a complete search. Our search terms were: "non-alcoholic fatty liver" or "NAFLD" or "non-alcoholic steatohepatitis" or "non-alcoholic fatty liver" or "non-alcoholic steatosis" or "non-alcoholic liver steatosis" or "non-alcoholic steatohepatitis" or "non-alcoholic steatosis" or "non-alcoholic hepatic steatosis" or "non-alcoholic liver steatosis" or "non-alcoholic hepatic steatosis" AND "black seed" or "Nigella sativa" or "black cumin" or "black seed oil" or "thymoquinone" AND "randomized controlled trial" or "controlled clinical trial" or "randomized" or "trial" or "placebo."

**Inclusion and exclusion criteria**

Researchers separately determined the eligibility of studies to be included in the review. Included studies were trials involving *N. sativa* seed powder or *N. sativa* seed oil versus placebo; *N. sativa* seed powder and lifestyle modification versus placebo and lifestyle modification; *N. sativa* combination use with another supplement versus placebo. Additional inclusion criteria were: (i) the study population included the patients with NAFLD and/or NASH; (ii) Supplementation of *N. sativa* seed powder or *N. sativa* seed oil for intervention; (iii) Hepatic steatosis (assessed by ultrasound or Fibroscan examination), liver enzymes, and histological indicators of inflammation and fibrosis had to be a primary or secondary outcome; (iv) the study was performed in subjects ≥ 18 years of age; and (v) published in English. Studies that included the participants in whom NAFLD was induced by excess alcohol intake, drugs, total parenteral nutrition, viruses, or genetic disorders, were excluded. In addition, study types such as systematic review, meta-analysis, narrative review, and animal studies were excluded. Bibliographic references of searched articles were also examined to find additional studies.

**Data items**

The data items that were considered in this study included: study design; country; sex; age; randomization and blinding; comparability of groups at baseline; types and doses of *N. sativa* supplementation; intervention protocol; duration of the study; assessment methods of hepatic steatosis; measures of liver enzymes; glucose metabolism biomarkers (FBG, serum insulin, and HOMA-IR), lipid profiles, blood pressure, inflammatory biomarkers, and anthropometric indices.

**Data extraction**

Authors independently performed data extraction from RCTs and then, discussed any differences in data extraction and eventually resolved them. Itemized Tables were applied to record relevant data from the reports. Results were converted to the same units to simplify the comparison among studies. In addition, the values of changes from baseline were converted to percentages.

**Study quality assessment**

Two authors (AM and AE) independently assessed the quality of selected studies using the Revised Cochrane Risk-of-Bias (RoB2) tool (Sterne et al., 2019 ▶). A third researcher (NS) judged any disagreement. This tool examines possible bias from randomization, deviation from intended intervention, measurement of outcome, missing outcome data, and the selection of reported results. The overall quality of each study was considered low risk of bias, some concerns of bias, or high-risk of bias. 

## Results

Initially, 28 articles were found by searching different databases. After removing duplicate reports, 22 studies were evaluated. Then, sixteen studies were excluded in the abstract screening stage because some did not meet the criteria for this study or the full texts were not accessible. 

**Figure 1:**
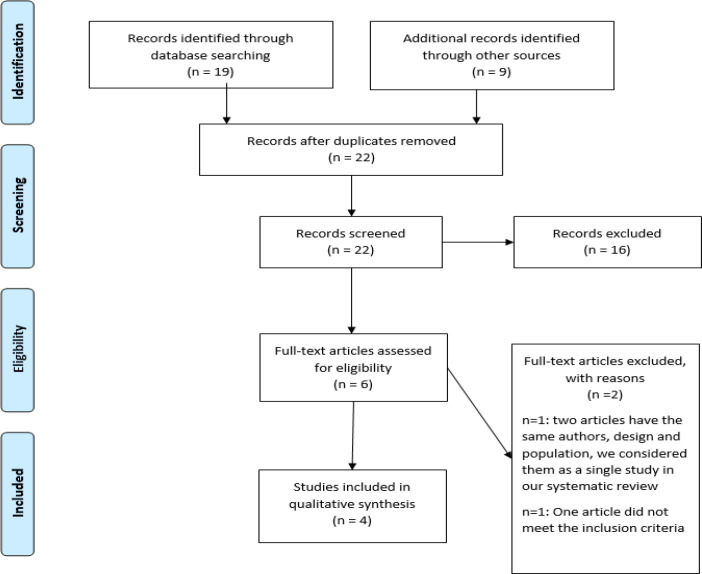
Flow diagram showing the process for inclusion of studies

Finally, the full texts were read for inclusion criteria and one study was excluded from the eligibility stage of this systematic review (Hosseini et al., 2018) (Figure 1). In the final judgment of RCTs, four studies with 324 patients were included (Darand et al., 2019a ▶; Darand et al., 2019b ▶; Hussain et al., 2017 ▶; Khonche et al., 2019 ▶; Rashidmayvan et al., 2019 ▶). Characteristics of included studies are summarized in Table 1.

**Table 1:** Studies that evaluated the effect of *N. sativa* supplementation on NAFLD outcomes

**Author, year**	**Country**	**Design**	**Total Sample Size (M/F)**	**Study population**	**Age (yrs)**	**BMI (Kg/m** ^2^ **)**	**Intervention and study groups**	**Duration(wks)**	**Outcomes**	**Results**
Darand et al., 2019 ▶ (24,25)	Iran	RCT	43 (21/22)	Patients with NAFLD	48.86±12.74	**G1**: 32.05± 4.17 **G2**: 31.7±3.54	**G1:** *N. sativa* seed powder (2 gr/d) +lifestyle modification **G2:** Placebo (rice starch) +lifestyle modification	12	ALT, AST, GGT, BMI, bodyweight, WC,WHR, hs-CRP,TNF-α,NF-κB, Fibrosis and steatosis grade (FibroScan), TG, HDL-C, LDL-C, TC, Serum glucose, Insulin, HOMA-IR, QUICKI	Steatosis ↓28% Serum glucose↓8%, serum insulin↓31%, HOMA‐IR↓36%, Steatosis % ↓ 28%, QUICKI↑9% TNF-α ↓20% Non-significant changes in lipid profile, liver enzymes, Fibrosis score
Khonche et al., 2019 ▶ (23)	Iran	RCT	120 (62/58)	Patients with NAFLD	46.64±12.18	27±2.1	**G1:** *N. sativa* seed oil (2.5 ml every 12 hr) **G2:** Placebo	12	AST, ALT, LDL-C, HDL-C, TG, Grade of hepatic steatosis(ultrasound)	ALT↓33%, AST↓30%, LDL-C↓15%, TG↓13%, HDL-C↑9% ↓ steatosis grade
Rashidmayvan et al., 2019 ▶ (26)	Iran	RCT	44 (29/15)	Patients with NAFLD	39±5.37	**G1**: 27.59±2.83 **G2**: 27.67±4.37	**G1:** *N. sativa* seed oil (1gr once a day) **G2:** Placebo (paraffin oil 1gr once a day)	8	SBP, DBP, FBS, TG, TC, HDL-C, LDL-C, VLDL-C, Insulin, AST, ALT, GGT, hs-CRP,TNF-α,IL-6,WC,WHR,BMI,body weight	AST↓30% , ALT↓19%, FBG↓7%, LDL↓18%, VLDL↓11%, TG↓19%, TC↓14%, HDL-C↑31% hs-CRP↓13%, , TNF-α↓20%, IL-6↓23% Non-significant changes in any of the outcomes' measures
Hussain et al., 2017 ▶ (22)	Pakistan	Randomized controlled trial	70 (44/26)	Patients with NAFLD	38±8.75	**G1**: 29.06±4.6 **G2**: 28.18±3.8	**G1:** *N. sativa* seed powder(1 gr twice/day) **G2:** Placebo (micro crystalline cellulose)	12	Body weight, BMI, ALT, AST, GGT, Fatty liver Grading(ultrasound)	ALT↓32%, AST↓32% ↓ Fatty liver Grading Body weight ↓11%, BMI↓9%, Non-significant Changes in GGT

Data are mean±standard deviation usually rounded to the nearest full figure; Sample size reflects those included in the final analysis. ALT, alanine aminotransferase; AST, aspartate aminotransferase ; BMI, body max index (kg/m2); d, day(s); DBP, diastolic blood pressure; F, female; FBG, fasting blood glucose; G1, group1; G2, group 2; GGT, gamma glutamyltransferase; h, hour; HOMA-IR, homeostasis model assessment; HDL-c, high density lipoprotein cholesterol; HS, hepatic steatosis; hs-CRP, high-sensitive C reactive protein; IL-6, interleukin 6; LDL-c, low density lipoprotein cholesterol; M, male; NAFLD, non-alcoholic fatty liver disease; NF-κB, Nuclear factor kappa B; QUICKI, quantitative insulin-sensitivity check index; RCT, randomized controlled trial; SBP, systolic blood pressure; TC, total cholesterol; TG, triglycerides; TNF-α, tumor necrosis factor -α ; TPM, Traditional Persian Medicine; VLDL, very low density lipoprotein; WC, waist circumference; WHR, waist to hip ratio

**Outcomes measurements**

**Effects of** ***N. sativa*** **on hepatic steatosis and liver enzymes**

The majority of reviewed RCTs had assessed the results of *N. sativa* supplementation on hepatic steatosis in patients with NAFLD. Two RCTs evaluated the change in fatty liver grade via ultrasound imaging (Hussain et al., 2017 ▶; Khonche et al., 2019 ▶). In one study, FibroScan was used to assess the amount of hepatic fibrosis and steatosis. This technique is a non-invasive and easy method to evaluate the stiffness of the liver (Darand et al., 2019a ▶). In three included studies, fatty liver and steatosis grades were reduced significantly after *N. sativa* supplementation (Darand et al., 2019a ▶; Hussain et al., 2017 ▶; Khonche et al., 2019 ▶). Also, serum levels of liver aminotransferases (AST and ALT) were measured in all RCTs. Serum concentrations of AST and ALT significantly reduced after *N. sativa* supplementation in three RCTs (Hussain et al., 2017 ▶; Khonche et al., 2019 ▶; Rashidmayvan et al., 2019 ▶). Gamma-glutamyltransferase (GGT) is another liver enzyme assessed in 3 trials, but no significant changes in any of them were observed (Darand et al., 2019b ▶; Hussain et al., 2017 ▶; Rashidmayvan et al., 2019 ▶).

**Effects of** ***N. sativa*** **supplementation on blood glucose, insulin resistance, and inflammatory factors**

Two RCTs evaluated the biomarkers of glucose metabolism, including serum glucose, serum insulin, HOMA-IR, and quantitative insulin sensitivity check index (QUICKI) (Darand et al., 2019a ▶; Rashidmayvan et al., 2019 ▶). Serum insulin and glucose were assessed in both studies (Darand et al., 2019a ▶; Rashidmayvan et al., 2019 ▶). Serum glucose was significantly decreased in both studies, but serum insulin was reduced significantly in Darand et al. study (Darand et al., 2019a). Also, in their trial, HOMA-IR and QUICKI were assessed; HOMA-IR significantly was decreased while QUICKI significantly was increased (Darand et al., 2019a ▶). 

Two RCTs evaluated the inflammatory biomarkers as secondary outcomes, including tumor necrosis factor α (TNF-α), high-sensitive C-reactive protein (hs-CRP), nuclear factor kappa-B (NF-κB), and interleukin-6 (IL-6) (Darand et al., 2019b ▶; Rashidmayvan et al., 2019 ▶). TNF-α was significantly reduced in both studies; however, hs-CRP was decreased in one of the mentioned trials (Rashidmayvan et al., 2019 ▶). Also, IL-6 was assessed in Rashidmayvan et al. study and the results showed a significant reduction in this outcome after *N. sativa* supplementation (Rashidmayvan et al., 2019 ▶).

**Effects of** ***N. sativa*** **supplementation on lipids, anthropometric indices, and blood pressure**

Three trials assessed serum lipid levels, including triglycerides (TG), total cholesterol (TC), high-density lipoprotein cholesterol (HDL-C), low-density lipoprotein cholesterol (LDL-C), and very-low-density lipoprotein (VLDL). TG, LDL-C, and HDL-C were evaluated in all three studies (Darand et al., 2019a ▶; Khonche et al., 2019 ▶; Rashidmayvan et al., 2019 ▶). Only in two of these studies, LDL-C and TG were significantly decreased, and HDL-C was increased considerably (Khonche et al., 2019 ▶; Rashidmayvan et al., 2019 ▶). Two studies evaluated TC (Darand et al., 2019a ▶; Rashidmayvan et al., 2019 ▶), but only in one of them, TC significantly decreased (Rashidmayvan et al., 2019 ▶). Also, VLDL was assessed in Rashidmayvan et al. study and the corresponding results showed a significant reduction in this outcome after *N. sativa* supplementation (Rashidmayvan et al., 2019 ▶). Some anthropometric indices such as body mass index (BMI), body weight, waist circumference (WC), and waist to hip ratio (WHR) were assessed in 3 trials (Darand et al., 2019a ▶; Hussain et al., 2017 ▶; Rashidmayvan et al., 2019 ▶). The body weight and BMI were changed significantly in Hussain et al. study (Hussain et al., 2017 ▶); however, no changes were seen in other studies. Rashidmayvan et al. assessed blood pressure in their research. However, they did not find any significant differences in systolic blood pressure (SBP) or diastolic blood pressure (DBP) after the intervention (Rashidmayvan et al., 2019 ▶).

**Confounding Variables**

Based on the articles studied in this review, it was identified that age, sex, BMI, WHR, physical activity, and dietary intakes are potential confounders in the effect of *N. sativa* on NAFLD. Two studies adjusted for age, sex, BMI (Hussain et al., 2017 ▶; Khonche et al., 2019 ▶), and one adjusted for BMI, WHR, physical activity, and dietary intakes (Darand et al., 2019b ▶).

**Quality assessment**

**Figure 2:**
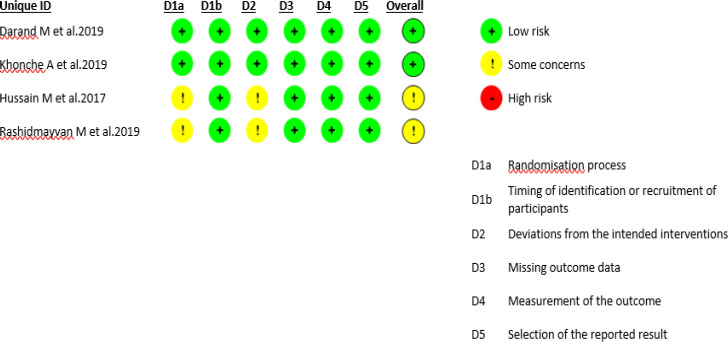
Risk of bias for each included study

The risks of bias in each domain are shown in Figure 2. Two trials had an overall score of "low risk" (Darand et al., 2019a ▶; Khonche et al., 2019 ▶) and the remaining two had an overall score of "some concern" (Hussain et al., 2017 ▶; Rashidmayvan et al., 2019 ▶). The randomization process and the deviation from the intended intervention were two domains that got scored as "some concern" in the trials by Hussain et al. and Rashidmayvan et al. (Hussain et al., 2017 ▶; Rashidmayvan et al., 2019 ▶). 

## Discussion

For many years*, N. sativa *has been known as a hepatoprotective herb (Darakhshan et al., 2015 ▶). One of the main active ingredients of *N. sativa* is thymoquinone (TQ) which has anti-oxidant, anti-inflammatory, and anti-fibrotic properties (Abbasnezhad et al., 2015 ▶; Salem, 2005 ▶). The suggested mechanisms of action of *N. sativa*, relevant to NAFLD, are summarized in Table 2. These rationales of the effects of *N. sativa* on the liver provided the basis for clinical trial studies. In recent years, several studies have been conducted in this field. Therefore, the primary purpose and innovation of the present study was to critically review the clinical trials on the effects of *N. sativa* on NAFLD, compare their findings and limitations in a single framework, and provide suggestions for a better design of future studies. In the following paragraphs, we discuss the results of the included studies in terms of the effect of *N. sativa* on hepatic steatosis, liver enzymes, insulin resistance, lipid profile, inflammatory factors, and BMI.

**Table 2:** Mechanisms related to hepatoprotective effects of *Nigella sativa*

Mechanism action of *N.sativa* on **liver enzymes**	**Increased** fatty acid beta-oxidation, mitochondrial function, and production of ATP **Decreased** oxidative stress in the liver cells, and lipid accumulation in the liver
Mechanism action of *N.sativa* on **obesity**	**Increased** basal metabolism **Decreased** insulin resistance, oxidative stress, lipid profile, and appetite
Mechanism action of *N.sativa* on **lipid profile**	**Increased** expression of LDL receptor genes **Decreased** LDL-C oxidation, Intestinal absorption of cholesterol, and HMGCR activity **Down-regulated** of APO-B100
Mechanism action of *N.sativa* on **serum glucose and insulin resistance**	**Increased** gene expression of GLUT-4, and AMPK activity **Decreased** Liver gluconeogenesis, and TNF-alpha
Mechanism action of *N.sativa* on **inflammation**	**Decreased** production of ROS, production of PG-E2, IL-6, AP-1 activity, TNF-alpha, and hs-CRP **Inhibiting** IRAK1, and NF-kB
*Mechanism effect of N.sativa* on **oxidative stress**	**Increased** glutathione level, catalase activity, SOD activity, and quinone reductase activity **Decreased** MDA, ROS, lipid peroxidase activity, and COX-1,2 activity
Mechanism action of *N.sativa* on **apoptosis**	**Increased** sirtuin 1 expression, B-cell lymphoma-2 level **Decreased** caspase 3 expression
Mechanism effect of* N.sativa* on **fibrosis**	**Increased** AMPK activity **Decreased** TGF-β, and α-smooth muscle actin expression

AMPK, AMP-activated protein kinase; AP-1, Activator protein 1; APO-B100, Apolipoprotein B-100; ATP, Adenosine triphosphate; COX, cyclo-oxygenase; GLUT-4,Glucose transporter type 4; HMGCR, 3-Hydroxy-3-Methylglutaryl-CoA Reductase; hs-CRP, high-sensitive C reactive protein; IL-6, interleukin 6; IRAK1, Interleukin 1 Receptor Associated Kinase 1; LDL-c, low density lipoprotein cholesterol; MDA, malondialdehyde; NF-κB, Nuclear factor kappa B ; PG-E2, Prostaglandin E2; ROS, Reactive oxygen species; SOD, Superoxide dismutase; TGF-β,Transforming growth factor beta; TNF-α, tumor necrosis factor–α

In three studies that assessed steatosis levels, the grade of fatty liver and steatosis were decreased significantly after *N. sativa* supplementation (Darand et al., 2019a ▶; Hussain et al., 2017 ▶; Khonche et al., 2019 ▶). In Khonche et al. study, 60 individuals with NAFLD consumed 2.5 ml fully standardized *N. sativa* seed oil every 12 hr, and 60 other patients consumed placebo in 3 months. At the end of the study, *N. sativa* seed oil significantly improved fatty liver grade as evaluated using ultrasound imaging. It has been suggested that monounsaturated fatty acids (MUFAs) and polyunsaturated fatty acids (PUFAs) may improve fatty liver disease by increasing fatty acid oxidation and inhibiting triglyceride synthesis in the liver (Silva Figueiredo et al., 2018 ▶). In fact, the essential oil of *N. sativa* has a higher content of PUFAs in comparison to MUFAs (Salehi et al., 2021 ▶). It seems that the possible roles of PUFAs on NAFLD may be more effective than MUFAs (Sheashea et al., 2021 ▶). Also, Khonche et al. study reported that *N. sativa* seed oil might protect against hepatic damage in NAFLD (Khonche et al., 2019 ▶). The study by Darand et al. showed a significant reduction in hepatic steatosis grade after the *N. sativa* supplementation (Darand et al., 2019a ▶). They conducted a 12-week trial in 50 NAFLD patients randomly assigned to receive two g/day of either *N. sativa* or placebo and lifestyle modification in both groups. Their results demonstrated that daily consumption of 2 g *N. sativa* supplementation and dietary recommendations are more effective in reducing steatosis in NAFLD patients than dietary recommendations alone. Using transient elastography for evaluating NAFLD in Darand et al. study differentiates their study from others. This method can identify and assess liver steatosis even if the amount of accumulated lipid is ten percent (Dowman et al., 2011 ▶). The amelioration of steatosis in their study may be related to the antioxidant properties of *N. sativa* and its lipid oxidation suppressive effect (Moschen et al., 2012 ▶). However, because of its potential risk in cardiovascular disease, only one dosage of *N. sativa* was given, and it was the main limitation of Darand et al. study. Of note, using different methods for monitoring liver steatosis and fibrosis would find conflicting results among the studies. In the present systematic review, three RCTs (Hussain et al., 2017 ▶; Khonche et al., 2019 ▶; Rashidmayvan et al., 2019 ▶) used ultrasound and one RCT (Darand et al., 2019a ▶) used transient elastography to identify and assess the fatty liver grade. It is essential to be noted that these methods cannot detect any histological changes in the liver as precisely as the biopsy method (Saleh and Abu-Rashed, 2007 ▶). Liver biopsy remains the gold-standard method to detect and evaluate fibrosis, steatosis, and inflammation in patients with NAFLD; however, it is restricted by cost, procedure-related morbidity and mortality, intra- and inter-observer variability, sampling error, and ethical issues (Chartampilas, 2018 ▶; Saleh and Abu-Rashed, 2007 ▶). 

Serum concentrations of liver enzymes (ALT, AST, and GGT) significantly reduced after *N. sativa* supplementation in three RCTs (Hussain et al., 2017 ▶; Khonche et al., 2019 ▶; Rashidmayvan et al., 2019 ▶). However, in Darand et al. study, no significant changes were observed in liver enzymes after the intervention (Darand et al., 2019a ▶). Failure to observe significant changes in liver enzyme levels after the intervention in their study may be due to the normal level of these enzymes at the study's baseline. The mitochondrial action enhancement is one of the mechanisms of TQ on lowering the liver enzymes levels (Sayed-Ahmed and Nagi, 2007 ▶).

NAFLD is associated with atherogenic dyslipidemia and HDL dysfunction (Katsiki et al., 2016 ▶). Of note, it has been proposed that *N. sativa* improves fatty acid beta‐oxidation in the liver (Balbaa et al., 2017 ▶; Hosseinian et al., 2018 ▶; Khaldi et al., 2018 ▶) (Table 2). Out of the three reviewed trials that evaluated lipid profile (Darand et al., 2019a ▶; Khonche et al., 2019 ▶; Rashidmayvan et al., 2019 ▶), two trials reported a significant change in lipid profile after *N. sativa* supplementation (Khonche et al., 2019 ▶; Rashidmayvan et al., 2019 ▶). Tocopherol, TQ, and phytosterol are antioxidant compounds in *N. sativa*, which suppress intestinal absorption of cholesterol, decrease the creation of hydroxyl–methyl–glutaryl coenzyme A reductase (HMGCR), inhibit LDL‐C oxidation, and increase the expression of LDL receptor genes via reduction of intracellular cholesterol ( Al-Naqeeb and Ismail, 2009 ▶; Brufau et al., 2008 ▶; Mariod et al., 2009 ▶). For these reasons, *N. sativa* may have a beneficial effect on lipid profile. However, at the end of the Darand et al. study, lipid profile, including total cholesterol, LDL‐C, and TG, did not significantly decrease in the group that received* N. sativa*. The normal serum lipid profile level at the onset of the trial was probably a reason for these unexpected outcomes (Darand et al., 2019a ▶).

One of the most important features of metabolic syndrome is insulin resistance, which is the common risk factor for NAFLD progression (Hamaguchi et al., 2005 ▶; Lomonaco et al., 2012 ▶; Pagano et al., 2002 ▶). Insulin improves glucose absorption for glycogen storage or glucose oxidation in the liver and muscle. The prominent role of insulin is to inhibit gluconeogenesis and glycogenolysis in the liver. In addition, insulin increases triglyceride and cholesterol synthesis in the liver (Biddinger et al., 2008 ▶). 

In the present study, some included RCTs assessed HOMA-IR and QUICKI (Darand et al., 2019a ▶; Rashidmayvan et al., 2019 ▶). Because of the good correlation between HOMA-IR and glycemic clamp, HOMA-IR is usually assumed as insulin resistance index in NAFLD patients (Salgado et al., 2010 ▶). HOMA-IR and QUICKI are used to detect insulin resistance in epidemiological and clinical studies (Hrebicek et al., 2002 ▶; Salgado et al., 2010 ▶). In Darand et al. study, HOMA-IR and QUICKI were improved significantly compared to the study's baseline values (Darand et al., 2019a ▶). Serum insulin and glucose were assessed in two included RCTs (Darand et al., 2019a ▶; Rashidmayvan et al., 2019 ▶). Serum glucose was significantly decreased in both mentioned trials, while serum insulin was significantly reduced only in Darand et al. study (Darand et al., 2019a ▶). The possible reasons for these conflicting results might be the differences in the amount and type of *N. sativa* used in the supplements and the duration of the intervention periods.

As previously mentioned, hypertension, as a cardiovascular disease (CVD) risk factor, is associated with NAFLD (Katsiki et al., 2016 ▶). Some studies have shown the association between high blood pressure and NAFLD (Catena et al., 2013 ▶; Fallo et al., 2008 ▶; Lopez-Suarez et al., 2011 ▶). Rashidmayvan et al. performed an 8-week randomized, double-blind, placebo-controlled clinical trial in 44 patients diagnosed with NAFLD. Patients were randomly divided into two groups; the intervention group consumed 1000 mg *N. sativa* oil and the other group consumed paraffin oil as a placebo (Rashidmayvan et al., 2019 ▶). Blood pressure was measured as a secondary outcome only in this included RCT. However, the corresponding results showed no change in SBP or DBP after *N. sativa* intake. Of note, based on the reported mean of the blood pressure, most of the participants were normotensive at baseline. The effects of *N.* *sativa* on blood pressure may be more pronounced in patients with hypertension. It seems that RCTs with a longer duration of intervention and higher doses of *N. sativa* on participants with hypertension, are needed to elucidate the effects of this supplement on blood pressure. 

Inflammation plays an important role in progressing from simple steatosis to steatohepatitis in NAFLD. In the present study, two included trials assessed the impact of *N. sativa* supplementation on inflammatory biomarkers (Darand et al., 2019a ▶; Rashidmayvan et al., 2019 ▶). TNF-α was significantly reduced in both studies; however, hs-CRP was decreased in one of the mentioned trials (Rashidmayvan et al., 2019 ▶). Also, IL-6 was assessed in Rashidmayvan et al. study and the results showed a significant reduction in this outcome after *N. sativa* supplementation (Rashidmayvan et al., 2019 ▶). None of the included trials evaluated the effect of *N. sativa* on pro-fibrotic biomarkers such as transforming growth factor-β (TGF-β) and procollagen III propeptide. Fibrosis is the advanced form of NAFLD that predisposes patients to liver cirrhosis (Sayiner et al., 2018 ▶). Pro-fibrotic factors activate the hepatic stellate cells (HSC) which are responsible for liver fibrosis (Gandhi, 2017 ▶). Interestingly, there is evidence that TQ may have beneficial effects on hepatic fibrosis via reducing mRNA expression of α-smooth muscle actin (α-SMA), collagen-І and tissue inhibitor of metalloproteinase-1 (TIMP-1) (Asgharzadeh et al., 2017 ▶; Bai et al., 2014 ▶). Therefore, designing clinical trial studies to investigate the effect of *N. sativa* on pro-fibrotic factors in patients with NAFLD is suggested.

Among the included trials, only in Hussain et al. study, body weight and BMI were changed significantly after the *N. sativa* supplementation (Hussain et al., 2017 ▶). However, the lack of assessment of changes in participants' energy and nutrient intake was one of the most important limitations of their study. In other words, the weight loss observed in that study may be due to a change in energy intake, not necessarily simply due to *N. sativa* consumption. Of the included studies, only Darand et al. evaluated participants' energy intake during the intervention (Darand et al., 2019a ▶). Their results showed that weight and BMI were decreased significantly only in the *N. sativa *group, whereas energy intake was reduced significantly in both groups (Darand et al., 2019a ▶). Because the changes in energy intake and physical activity were not different between the two groups at the end of the trial, they concluded that *N. sativa *might decrease body weight through rising basal metabolism (Darand et al., 2019a ▶). The suggested mechanisms of the effects of *N. sativa* on body weight are shown in Table 2. The RCTs included in our systematic review had different study durations and amounts of *N. sativa* supplementation. As a result, some contradictory results exist between them. More RCTs are needed to confirm the proper doses and intervention periods of *N. sativa* supplementation on NAFLD. In addition, different types of *N. sativa* were used in the reviewed studies. Darand et al. and Hussain et al. used *N. sativa* seed powder (Darand et al., 2019a ▶; Hussain et al., 2017 ▶), while Khonche et al. and Rashidmayvan et al. used *N. sativa* seed oil (Khonche et al., 2019 ▶; Rashidmayvan et al., 2019 ▶). It should be noted that TQ, as an active ingredient of *N. sativa*, has a limited bioavailability. Since it is a hydrophobic agent, it is more available in the essential oil of *N. sativa* compared to the whole seeds (Goyal et al., 2017 ▶). On the other hand, in the blood, TQ is carried by the human serum albumin (HAS) and α1-acid glycoprotein (AGP) (Lupidi et al., 2012 ▶). Under normal physiological conditions, TQ preferentially binds to HAS. However, because blood levels of AGP are increased considerably in inflammatory diseases, such as NAFLD, the interaction of TQ with AGP may affect the pharmacokinetics and pharmacodynamics of TQ (Lupidi et al., 2012 ▶). Today, efforts are being made to increase the bioavailability of TQ using pharmaceutical nanotechnology (Goyal et al., 2017 ▶). For more information about the pharmacokinetics and pharmacodynamics of TQ and *N. sativa*, please refer to Goyal et al. (Goyal et al., 2017 ▶). Despite all mentioned above, it should be noted that the consumption of *N. sativa* whole seeds can provide fiber and some vitamins and minerals that may have beneficial effects on the metabolic profiles of patients with NAFLD.

When RCTs were reviewed, two studies were identified from the same authors that presented data from the same subjects (Darand et al., 2019a ▶; Darand et al., 2019b ▶). They were considered a single study in our systematic review to reduce possible errors, although we reported all outcomes (Darand et al., 2019a ▶; Darand et al., 2019b ▶). The limitation of the present study was the ineffectiveness of conducting a meta-analysis of the included data. Because of the few studies conducted to evaluate the effects of *N. sativa* on NAFLD, their heterogeneity would be high and meta-analysis was not possible. In other words, if significant heterogeneity is detected among the studies, including their data in a meta-analysis study will yield inconclusive findings (Higgins et al., 2003 ▶). As a result, we did not conduct a meta-analysis on studies' data. Recently, a systematic review and meta-analysis has been conducted on the effects of *N. sativa* in patients with NAFLD and has assessed the relationship between *N. sativa*, lipid profile, glycemic parameters, liver enzymes, inflammatory factors, and grade of fatty liver (Tang et al., 2021 ▶). The study indicated that AST, ALT, FBS, HDL, and hs-CRP were improved after *N. sativa* supplementation in patients with NAFLD, but there were no significant changes in TC, LDL, TG, insulin, and TNF-α. However, as mentioned and predicted, the heterogeneity index in this meta-analysis was high, limiting the conclusion about the definitive effects of *N. sativa* on NAFLD outcomes.

The strength of our study was the extensive literature review and adherence to the PRISMA statement. In addition, one of the important aims of the present study was providing suggestions that help designing future studies to better clarify the beneficial roles of *N. sativa* in NAFLD prevention and treatment. Based on the results of the clinical trials reviewed in the present study, we would suggest the following points:

1. More accurate and precise measurement methods to monitor liver histopathology (e.g. liver biopsy, magnetic resonance spectroscopy, or transient elastography) should be used.
2. It would be better to divide the study groups into different subtypes of steatosis, steatohepatitis, and liver fibrosis and then compare the effects of *N. sativa* supplementation between them.
3. Studies with the specific objective to evaluate the combination therapy of *N. sativa* with other herbal or nutritional supplements to investigate their synergistic effects on NAFLD are suggested.
4. Assessing the effects of *N. sativa* supplementation on serum biomarkers of inflammation, oxidative stress, and pro-fibrotic factors would be more inclusive.
5. Investigation about the gene expression of each biomarker of NAFLD after *N. sativa* supplementation would bring advanced results.
6. The use of nano-encapsulated forms of TQ in future interventions on NAFLD will reveal valuable evidence.

Although the efficacy of *N. sativa* on liver enzymes and the grade of hepatic steatosis has been found in some of the included studies, more well-designed clinical trials are needed to determine the definitive effects of *N. sativa* on NAFLD.

## Conflicts of interest

The authors have declared that there is no conflict of interest.
